# Editorial: Epigenetic Mechanisms Regulating Neural Plasticity

**DOI:** 10.3389/fncel.2019.00118

**Published:** 2019-04-24

**Authors:** Daniel Ortuño-Sahagún, Reinhard Schliebs, Merce Pallàs

**Affiliations:** ^1^Laboratorio de Neuroinmunomodulación Molecular, Instituto de Investigación en Ciencias Biomédicas, Centro Universitario de Ciencias de las Salud, Universidad de Guadalajara, Guadalajara, Mexico; ^2^Medical Faculty, Paul Flechsig Institute for Brain Research, University of Leipzig, Leipzig, Germany; ^3^Department of Pharmacology and Therapeutic Chemistry, Institut de Neurociències, University of Barcelona, Barcelona, Spain

**Keywords:** neural plasticity, epigenetics (MeSH), aging, epigenetic regulating mechanisms, central nervous system, epigenome, neurodegenerative disease, CNS disorders

While larger parts of the genome have been deciphered and sequenced during the last decade, it is now possible to identify practically all the genes that a cell or tissue transcribes and translates at a given moment in time (Wheeler et al., 2008). Thus, the challenge we are now facing is to understand the epigenetic regulation of all this information, it will be the next great task in the near future. The central nervous system (CNS), and especially the brain, has evolved to make humans the way they are, and it is strongly influenced and affected by epigenetic factors (Stroud et al., 2017). Indeed, many extrinsic factors can alter neural activity during the lifetime of an organism (from the embryo to adult and during aging), including diet, exercise, environmental variations, stressors, etc. These stimuli exert their effect through processes commonly referred to as “Epigenetic mechanisms” (Sen et al., 2016), which mainly involve DNA methylation and demethylation, protein acetylation and deacetylation (mainly histones) and regulatory small non-coding RNAs, primarily microRNAs (miRNA) activity, all important events in the regulation of neuronal plasticity. Consequently, this special issue aims to shed light on the physiological and pathological processes affected by epigenetic mechanisms that are involved in the regulation of neural plasticity, highlighting the potential diagnostic and therapeutic strategies that may become commonplace in the future. Accordingly, this volume presents a collection of works, both reviews and original studies, that address the aforementioned issues, representing the ideas of 100 researchers from eight countries on three continents.

Epigenetic mechanisms have been proposed to strongly participate in the adaptive features of the CNS, both those of a physiological (from embryonic development through aging) and pathological (from cancer to neurodegenerative conditions) nature. Changes in the epigenome allow neurons to reorganize in response to intrinsic or extrinsic stimuli, and to develop or maintain specific activities. As such, the study of epigenetics is potentially interesting when attempting to develop strategies that modulate changes provoked in pathological conditions, particularly since extrinsic factors and pharmacological tools can modify neural function in an organism throughout its life. Therefore, diet, exercise, environmental conditions, stressors or drugs can alter pathological events to benefit the patient, driving epigenetic processes that co-ordinate a number of translational processes.

Post-translational modifications have been implicated in neurological pathologies like Parkinson's disease (PD), including protein acetylation. In fact, when acetylated proteins and peptides were characterized in primary fibroblasts from patients with inherited and idiopathic PD, differences were described in the hyperacetylated and hypoacetylated peptides between the two pathological entities (see Yakhine-Diop et al.). Additionally, acetyl-CoA metabolism is relevant in regulating multiple protein acetylation reactions in the brain, which in turn regulate the functional and adaptative properties of brain cells (both neuronal and non-neuronal). Ronowska et al. highlights the importance of the changes in intraneuronal acetyl-CoA and its compartmentalization, which contributes to the development of different neurodegenerative brain disorders.

Epigenetic elements or tags can be read (recognized), written (established), and erased (removed) in nucleosomes, mainly through DNA methylation and demethylation. The influence of demethylation in the adult brain and on neurological disorders, as well as its possible functional consequences for health and disease, has been reviewed by Bayraktar and Kreutz. Similarly, a key role for epigenetic DNA modifications has been identified by Andrés-Benito et al. demonstrating a decrease in the methylation of the Katanin Interactor Protein gene (KIAA0566) in association with age and the presence of neurofibrillary tangles, the main pathological characteristics of taupathies. Indeed, this particular alteration in DNA methylation has important implications for the regulation of microtubule homeostasis in locus coeruleus neurons. The review by Penas and Navarro focuses on the implication of a wider set of epigenetic changes that appear to trigger modifications in nociception after neural lesions, including histone modifications, DNA methylation, the expression of non-coding RNAs and altered chromatin modifier activity. As such, novel putative targets to manage neuropathic pain are proposed, which remains an unmet clinical need, such as histone deacetylases (HDAC1, Sirt1), histone demethylases (JMJD3), histone methyltransferases (G9a, EZH2), DNA methyltransferase (DNMT3b), transcriptional repressor complex (REST), Methyl CpG binding protein (MeCP2), and various non-coding RNAs. The correct delivery of microRNAs is a key factor for their possible therapeutic application and Reza-Zaldivar et al. explain why exosomes could constitute an adequate vehicle for the delivery of microRNAs in biological systems, for example, as an alternative treatment for Alzheimer's disease.

In addition to the aforementioned epigenetic mechanisms, stress, changes in diet and the environment, or even situations of extreme environments like outer space, can influence neuroplasticity and consequently, affect the activity of the CNS. In this respect, Herrera et al. demonstrate that controlling dietary long-chain polyunsaturated fatty acid (L-CPFA) content modulates the expression of synaptogenesis-related neuronal proteins, in conjunction with the influence of hormones. Indeed, estradiol and progesterone levels, as well as cyclic ovarian secretory activity, may modulate the effects of dietary L-CPFA in cerebral cortex physiology, which for example, may have clinical implications for postmenopausal women. In addition, Benoit et al. show how nutrient variations and hormonal changes early in development can reprogram metabolism in adulthood. Specifically, they demonstrate the changes in expression of eleven microRNAs induced by hyperinsulinemia and/or hyperleptinemia in the arcuate nucleus, a master regulator of energy homeostasis. This reveals how an altered perinatal environment can affect metabolism in adulthood.

With respect to the importance of the environment on genomic expression, Griñán-Ferré et al. demonstrate how significant improvements in brain function and cognitive abilities can be achieved in a model of aging through interventions that involve environmental enrichment. Importantly, these authors demonstrate that epigenetic mechanisms at least in part drive some of the beneficial effects of environmental enrichment, such as the decreased neuroinflammation and enhanced synaptic function. As described previously, environmental enrichment increases the cognitive reserve in humans, helping combat the deleterious effects of neurodegeneration (Redolat and Mesa-Gresa, 2012). Hence, the individual's physical and social surroundings are clearly important in providing the brain with some arms to resist neurodegenerative processes, increasing resilience of paramount importance in aging processes.

By contrast, the work by Muñoz-Llanos et al. focuses on the effect of chronic stress on synaptic plasticity in the hippocampus, giving importance to the effect of epigenetic mechanisms on learning and memory processes. They identified miRNA-92a and miR-485 as potential targets to produce changes in gene regulatory networks within the hippocampus of stressed rats, modifying the dynamics of neuroplasticity. As such, these non-coding regulatory RNAs could represent novel targets for the treatment of depression.

Finally, Cui et al. present a detailed genomic analysis of the differentiation of neuronal stem cells (NSCs) in outer space flights. During such spaceflight experimental conditions, NSCs maintain a greater capacity for stemness even though their growth rate slows down. This type of study will help us begin to understand the possible physiological changes in the CNS associated with future space travel.

In conjunction, in this volume breakthrough new information is collected regarding the cellular and molecular mechanisms underlying synaptic plasticity in neurodegenerative diseases like Parkinson's, or Alzheimer's, as well as in other CNS disorders like depression or neuropathic pain. In addition, further valuable information is provided regarding the physiology of the brain, all from the perspective of epigenetic regulation.

## Author Contributions

All authors listed have made a substantial, direct and intellectual contribution to the work, and approved it for publication.

### Conflict of Interest Statement

The authors declare that the research was conducted in the absence of any commercial or financial relationships that could be construed as a potential conflict of interest.
